# An Indole Alkaloid Extracted from *Evodia rutaecarpa* Inhibits Colonic Motility of Rats *In Vitro*

**DOI:** 10.1155/2020/8610653

**Published:** 2020-04-03

**Authors:** Guo-xiang Wang, Yan-li Xiang, Hong-gang Wang, Yang-de Miu, Guang Yu

**Affiliations:** Department of Gastroenterology, Taizhou Municipal Hospital, Taizhou, Zhejiang, China 318000

## Abstract

Evodiamine (Evo) is an indole alkaloid extracted from the traditional Chinese medicinal herb *Evodia rutaecarpa*. Evo may regulate gastrointestinal motility, but the evidence is insufficient, and the mechanisms remain unknown. The aim of this study was to investigate the effect of Evo on colonic motility of rats and the underlying mechanisms *in vitro*. Rat colonic muscle was exposed to Evo (10 and 100 *μ*M) followed by immunohistochemistry of cholecystokinin receptor 1 (CCK1R). Muscle contractions were studied in an organ bath system to determine whether CCK1R, nitric oxide (NO), and enteric neurons are involved in the relaxant effect of Evo. Whole-cell patch-clamp was used to detect L-type calcium currents (*I*_Ca,L_) in isolated colonic smooth muscle cells (SMCs). CCK1R was observed in SMCs, intermuscular neurons, and mucosa of rat colon. Evo could inhibit spontaneous muscle contractions; NO synthase, inhibitor L-NAME CCK1R antagonist, could partly block this effect, while the enteric neurons may not play a major role. Evo inhibited the peak *I*_Ca,L_ in colonic SMCs at a membrane potential of 0 mV. The current-voltage (I–V) relationship of L-type calcium channels was modified by Evo, while the peak of the I–V curve remained at 0 mV. Furthermore, Evo inhibited the activation of L-type calcium channels and decreased the peak *I*_Ca,L_. The relaxant effect of Evo on colonic muscle is associated with the inhibition of L-type calcium channels. The enteric neurons, NO, and CCK1R may be partly related to the inhibitory effect of Evo on colonic motility. This study provides the first evidence that evodiamine can regulate colonic motility in rats by mediating calcium homeostasis in smooth muscle cells. These data form a theoretical basis for the clinical application of evodiamine for treatment of gastrointestinal motility diseases.

## 1. Introduction

Evodiamine (Evo), a naturally occurring indole alkaloid, is one of the main bioactive components of *Evodia rutaecarpa*. It has been used for several hundred years as traditional Chinese medicine and was first recorded in the Shennong Herbal Classic. Concerning the pharmacological action of Evo, more attention has been paid to its beneficial effects on cancer, obesity, nociception, inflammation, cardiovascular diseases, Alzheimer's disease, infectious diseases, and thermoregulation [1–7]. Recent studies have demonstrated that Evo may be involved in regulating gastrointestinal motility, but the evidence is insufficient, and the potential mechanisms remain unknown.

A previous study showed that Evo inhibited gastrointestinal transit and gastric emptying by activating gastrointestinal cholecystokinin receptor 1 (CCK1R) [8]. Another study showed that Evo enhanced the contractility of jejunal smooth muscle, which is calcium-dependent and requires mediation by endogenous acetylcholine [9]; Cajal interstitial cells are also involved. In addition, the expression of myosin light chain kinase is increased. The enteric nervous system, which can still perform its main regulatory role *in vitro*, regulates intestinal motility, indicating that isolated intestinal fragments can be used to study the effect and mechanism of drugs [10].

Dysfunction of gastrointestinal motility and abdominal pain are the main characteristics of functional gastrointestinal diseases. Many studies have shown that the mechanism is related to abnormal changes in smooth muscle and the sensory system, which govern normal gastrointestinal motility. In this study, we explored the effect of Evo on colonic motility in rats and the underlying mechanisms, aiming to provide a theoretical basis for its application in the clinical treatment of gastrointestinal motility diseases.

## 2. Materials and Methods

We followed the methods of Lu et al. [11] and Quan et al. [12].

### 2.1. Animals

All experimental animal procedures were approved by the Institutional Animal Care and Use Committee and adhered to the ethical guidelines of the International Association for the Study of Pain. Male Wistar rats weighing 180–220 g were used for this study. The animals were housed in a facility with constant temperature (20–24°C) and 65% humidity and provided with food and water *ad libitum*.

### 2.2. Solutions and Reagents

Calcium-free physiological salt solution (PSS) was prepared as follows (mmol/L): sodium chloride 135.0, potassium chloride 5.0, magnesium chloride 21.2, glucose 10.0, and hydroxyethyl piperazine ethylsulphonic acid 10.0, with sodium hydroxide to adjust the pH to 7.4. L-type calcium channel electrode solution was prepared as follows (mmol/L): caesium chloride 135, magnesium chloride 24, hydroxyethyl piperazine ethylsulphonic acid 10, anhydrous creatine phosphate disodium salt 2, ethylene glycol-bis-(2-aminoethyl ether) tetraacetic acid 10, and tetraethylamine 20, with caesium hydroxide to adjust the pH to 7.3. Tyrode's solution was prepared as follows (mmol/L): sodium chloride 147.0, potassium chloride 4.0, calcium chloride 2.0, sodium dihydrogen phosphate 0.42, disodium hydrogen phosphate 2.0, magnesium chloride 1.05, and glucose 5.5, with sodium hydroxide to adjust the pH to 7.4. Evo was purchased from Aladdin Reagent Co., Ltd. Evodiamine was dissolved in dimethyl sulphoxide (DMSO). DMSO was ineffective per se at the volumes employed in this study. Tetrodotoxin (TTX), L-NAME, and CCK1R antibody were suspended in calcium-free PSS.

### 2.3. Immunohistochemistry

The localization of CCK1R protein was examined in the distal colon by immunohistochemistry. Formalin-fixed tissues were embedded in paraffin and cut into 5 *μ*m thick sections. Following antigen unmasking, tissue sections were incubated overnight at 4°C with rat anti-CCK1R antibody (1 : 100). After repeated washing with phosphate-buffered saline (PBS), the sections were incubated for 1 h at room temperature with an anti-rat secondary antibody in PBS/Triton, washed again, and finally incubated with streptavidin-peroxidase complex for another 20 min at room temperature. Diaminobenzidine was used as a chromogen, and hematoxylin was used for counterstaining.

### 2.4. Spontaneous Contraction of Colonic Smooth Muscle Strips

After being sacrificed, the distal colon was quickly removed in 3 cm pieces and placed in calcium-free PSS solution in a 95% O_2_ and 5% CO_2_ atmosphere. Under an anatomical microscope, the mucosa and submucosa were slightly dissected. Two muscle strips, of which the width was about 3 mm and the length about 8 mm, were taken along the direction of the smooth muscle fibers, including a longitudinal muscle (LM) strip from near the end of the colon and an LM strip from the distal end of the colon. The muscle strips were placed in two perfusion grooves. One end of each strip was fixed on the glass hook at the bottom of the groove, and the other was connected to a tension transducer. The signal was inputted into a physiological recorder to record the spontaneous contraction of the strips, which were incubated for about 20 min at 1.5 g preload. When the spontaneous contraction of the muscle strips was stable, the corresponding concentration of Evo was added.

### 2.5. Whole-Cell Patch-Clamp Test

After the rats were sacrificed, the distal colon was quickly removed and placed in oxygen-saturated calcium-free PSS. The mucosa and submucosa were slightly peeled off under the anatomical microscope. Then, the muscle strips were cut into small cubes about 2 mm × 3 mm in size and placed in digestive juice warmed to 37°C for 20–30 min. After the digestion period, the supernatant was discarded, and the segments were washed four times with Ca^2+^-free PSS to remove the enzyme. Single SMCs were dispersed by gentle trituration with a fire-polished Pasteur pipette and stored at 4°C. Suspensions of cells were dropped into a perfusion chamber that was mounted with an inverted microscope (Olympus, Japan) and allowed to adhere to the bottom of the chamber. After 10 min, the chamber was infused with Tyrode's buffer (1 mL/min). The whole-cell patch-clamp technique was used to record the L-type calcium currents with an Axopatch™ 700B amplifier (Axon Instruments, Burlingame, CA, USA). A patch pipette was made using a micropipette puller (P97; Sutter, Novato, CA, USA) and had a resistance of 4–6 M*Ω*. The data were digitized at 1 kHz and filtered at 800 Hz. All experiments were conducted at room temperature (22–25°C).

### 2.6. Statistical Analysis

Data were analyzed with SPSS 17.0 and pClamp 10.2 and expressed as the mean ± SEM. Significant differences between groups were evaluated using paired or unpaired Student's *t*-tests. The average amplitude during the 5 min period before treatment with Evo was measured as the baseline. In the groups pretreated with L-NAME, anti-CCK1R antibody, and TTX, the baseline was the average amplitude after the administration of L-NAME, anti-CCK1R antibody, and TTX. The average peak contraction measured as the amplitude of contraction above the basal level in the 3 min after each Evo treatment was normalized to a standardized ratio (R), in which the baseline of each experiment was equal to 1 (R = response maximal value/baseline). *P* value significance was set at 0.05.

## 3. Results

### 3.1. Immunohistochemical Localization of CCK1R in the Distal Colon

**Figure 1:**
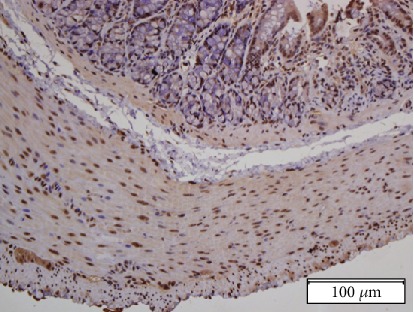
Immunohistochemical localisation of CCK1R in the rat distal colon.

To determine the expression of CCK1R in the distal colon of rats, immunohistochemistry was used. As shown in Figure 1, CCK1R was expressed in longitudinal and circular muscle cells, intermuscular neurons, and mucous membranes.

### 3.2. Effect of Evo on the Contractile Activity of Colonic Strips

**Figure 2:**
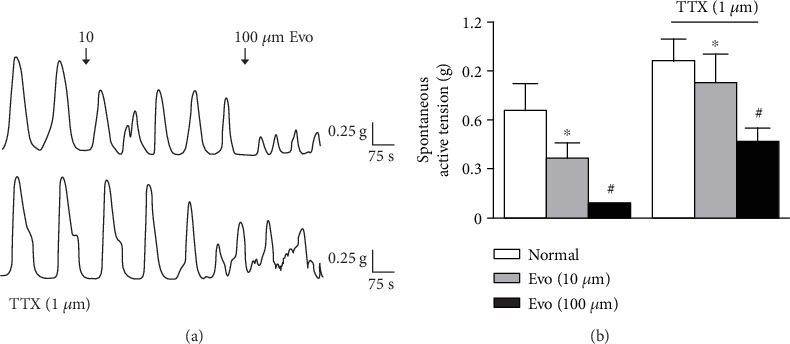
Effect of Evo on spontaneous contraction of colonic muscle strips. (a) Evo inhibited the spontaneous contractions of longitudinal muscle (LM), which was still recorded in the presence of TTX (1 *μ*M). (b) Summarised results of LM before and after application of Evo in the presence and absence of TTX (*n* = 7 for each group, ^∗^*P* < 0.05, ^#^*P* < 0.01 vs. control).

Evo inhibited spontaneous contractions of the colonic LM strips from the normal rats. As shown in Figure 2, Evo significantly reduced the baseline amplitude of spontaneous contractions of LM. The mean contractile amplitude before the addition of Evo was 0.67 ± 0.15 g. After the addition of Evo (10 and 100 *μ*M), the amplitude was reduced to 0.36 ± 0.09 g (*P* < 0.05 vs. control) and 0.10 ± 0.03 g (*P* < 0.01 vs. control), respectively. In the presence of TTX (1 *μ*M), the spontaneous contractions of LM increased, but the inhibitory effects of Evo were still present.

### 3.3. Effect of Evo on Nitric Oxide Synthase (NOS) and CCK1R in Longitudinal Smooth Muscle Strips of the Distal Colon

**Figure 3:**
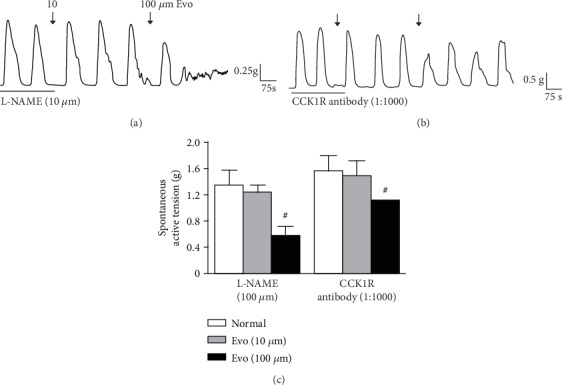
Effect of Evo on spontaneous contraction of colonic muscle strips. (a) Evo inhibited the spontaneous contractions of longitudinal muscle (LM) in the presence of L-NAME (10 *μ*M). (b) Evo inhibited the spontaneous contractions of longitudinal muscle (LM) in the presence of CCK1R antibody (1 : 1000). (c) Summarised results of the LM contractile activities when incubated with L-NAME or CCK1R antibody. The values are expressed as the mean ± SEM (*n* = 8 rats/group). Evo (100 *μ*M) decreased the spontaneous contraction of LM strips after incubation with either L-NAME or CCK1R antibody (^#^*P* < 0.01 compared with control).

As shown in Figure 3, after pretreatment with L-NAME (10 *μ*M) or CCK1R antibody (1 : 1000) for 10–20 min and subsequent application of Evo (10 *μ*M), the *R*-value of the LM strips decreased from 1.35 ± 0.24 and 1.58 ± 0.24 to 1.25 ± 0.13 and 1.50 ± 0.22, respectively (*P* > 0.05 vs. control). With the subsequent application of 100 *μ*M Evo, the *R*-value of the LM strips decreased to 0.59 ± 0.15 and 1.13 ± 0.15, respectively (*P* < 0.01 vs. control).

### 3.4. Evo Inhibited *I*_Ca,L_ in Colonic SMCs

**Figure 4:**
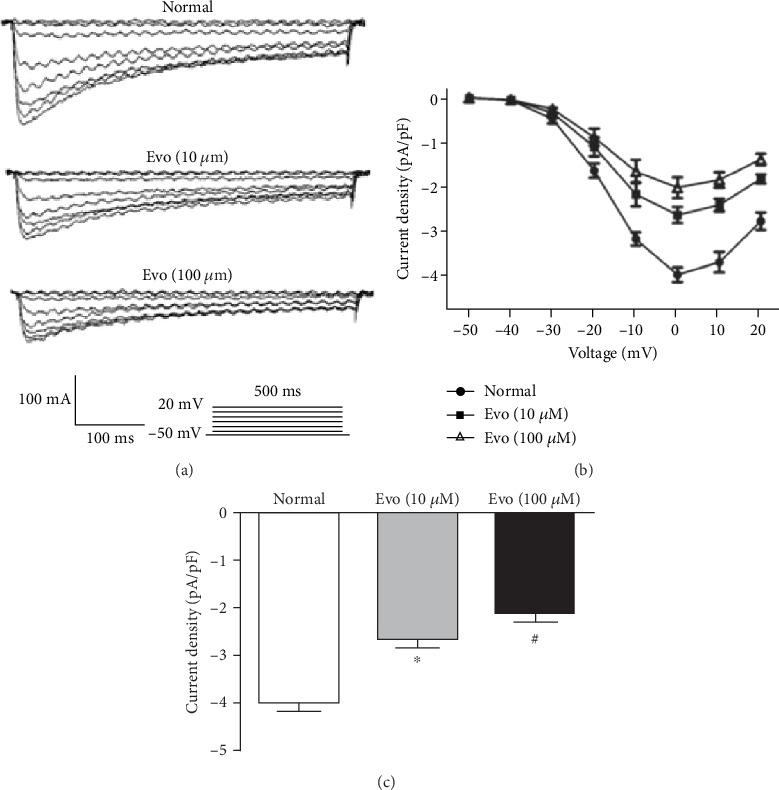
Effect of Evo on *I*_Ca_ in colonic SMCs. (a) Original traces of whole-cell recordings in response to a series of depolarising voltage pulses from a holding potential of -50 mV to +20 mV in 10 mV steps before (control) and after the application of Evo (10 and 100 *μ*M). (b) The representative effects of Evo at different concentrations on the I-V relationship of *I*_Ca_. (c) Summarised data showing the density of the currents at 0 mV (*n* = 5 for each group, ^∗^*P* < 0.05, ^#^*P* < 0.01 vs. control).

As shown in Figure 4, *I*_Ca,L_ was elicited by 10 mV depolarizing steps from a constant holding potential of -50 mV to +20 mV for 500 ms based on whole-cell voltage-clamp recordings. Bath application of Evo (10 and 100 *μ*M) caused suppression of the peak of *I*_Ca,L_. The *I*_Ca,L_ density was decreased successively from −3.92 ± 0.15 pA/pF (control) to −2.62 ± 0.19 pA/pF (Evo 10 *μ*M) and −2.07 ± 0.23 pA/pF (Evo 100 *μ*M) (*P* < 0.05 vs. control).

## 4. Discussion

In traditional Chinese medicine, Evo is often used as an analgesic, antiemetic, astringent, and antihypertensive drug [13]. Recently, many studies demonstrated that Evo may be a potential therapeutic drug against other chronic diseases such as renal tubulo-interstitial fibrosis [14], atherogenesis [15], hypoxia [16], glioma [17], hypomotility disorders [9], and IgE-induced allergenic diseases, including atopic dermatitis and rhinitis [18]. Moreover, Xiong et al. [9] reported that Evo exerted stimulatory effects on rat jejunal contractility, exhibiting its potential role in relieving hypomotility disorders. Furthermore, Cao et al. demonstrated that Evo reduced 24 h fecal weight in rats and inhibited the motility of colonic strips. Cholecystokinin (CCK) and CCK1R are thought to play a role in these effects [19]. The present study evaluated the expression of CCK1R in the rat colon, the effect of Evo on the contractile activity of colonic strips, and the effect of Evo on L-type calcium channels in colonic SMCs. We found that CCK1R is expressed in SMCs, intermuscular neurons, and mucosa of rat colon. In addition, Evo significantly inhibited the contractile activity of colonic LM strips, and this effect was partially blocked by anti-CCK1R antibody and L-NAME. Evo also inhibited L-type calcium channels in SMCs. These data provide the first evidence that Evo plays an inhibitory role in the regulation of L-type calcium channels, suggesting the involvement of Evo in the regulation of calcium homeostasis in the smooth muscle of the rat colon.

The gastrointestinal tract is a complicated system. Many factors, including nerves, neurotransmitters, neuropeptides, and hormones, are involved in regulating its functions and activities [20, 21]. It was reported that CCK, a member of the regulatory peptide family, may play an important role in regulating the physiological functions of the digestive tract, mainly acting on the receptors distributed therein. CCKR is a type of G protein-coupled receptor and includes CCK1R and CCK2R. CCK1R is primarily expressed in peripheral tissues, while CCK2R is mainly expressed in the central nervous system [22–25]. CCK1R expression in the digestive tract plays a role in mediating gallbladder contraction, pyloric sphincter relaxation, pancreatic growth, and pancreatic enzyme secretion and inhibits gastric emptying and gastric acid secretion [25]. Previous studies have shown that Evo may act on CCK-releasing peptide (CCK-RP) in the digestive tract, which then stimulates the secretion of CCK from duodenal cells, resulting in the release of CCK into the blood. Blood CCK acts on the CCK1R expressed in the gastrointestinal tract, thereby inhibiting gastric emptying and intestinal motility [8]. In our study, we found that the expression of CCK1R was observed in SMCs, intermuscular neurons, and mucosa of rat colon, and anti-CCK1R antibody could partly block the effect of Evo in inhibiting contractions of rat colon in vitro. This demonstrate that CCK1R may be partly related to the inhibitory effect of Evo on colonic motility. There may be other molecules involved in the inhibitory effect.

Nitric oxide (NO) is an essential nonadrenergic, noncholinergic (NANC) neurotransmitter in the enteric nervous system (ENS). It is generated from L-arginine by neuronal NOS (nNOS/NOS-I). NO has a potent inhibitory effect on the smooth muscle in several regions of the gastrointestinal tract [26]. Furthermore, NO is involved in a vast array of physiological functions, such as neurotransmission, vasodilation, and gastrointestinal motility [27, 28]. Several lines of evidence suggest that impairment of NO synthesis is associated with an altered antioxidant mechanism and gastrointestinal motility disorders [29, 30]. In rabbit, NO was shown to decrease spontaneous contractions in both smooth muscle layers [31]. Smith and Muir [32] demonstrated that NO enhances the frequency and amplitude of spontaneous electrical activity and increases the rate of contraction in the rabbit colon in vitro. NO production is mediated through the action of the enzyme NOS, which is highly localized in both neural and vascular elements of the rat small intestinal wall [33]. To examine whether NO was involved in the inhibitory effect of Evo on colonic motility, we treated the strips with the NOS inhibitor L-NAME. The results showed that the inhibitory effect of low-concentration Evo (10 *μ*M) was blocked by pretreatment with 10 *μ*M L-NAME, whereas the inhibitory effect of high-concentration Evo (100 *μ*M) was partly blocked. This result indicated that the effect of L-NAME blocking the inhibitory effect of Evo on the contraction of the gut may be reversible.

Furthermore, to examine whether enteric neurons was involved in this effect, we treated the strips with TTX. The results showed that the inhibitory effects of both low (10 *μ*M) and high-concentration Evo (100 *μ*M) were still present when being pretreated with 1 *μ*M TTX. Thus, the enteric nerve may be not majorly involved in the inhibitory action of Evo on gut motility, while CCK1R and NOS may play a major role.

It is known that increased [Ca^2+^]_I_, which leads to binding to calmodulin and activation of myosin light chain kinase, is the primary stimulus for contraction [34]. L-type calcium channel is the main ion channel regulating gastrointestinal smooth muscle contraction. The opening of calcium channels increases the influx of calcium ions and depolarizes cell membranes, thereby promoting smooth muscle contraction. The upregulation of L-type calcium channel expression in the colon is associated with colonic dyskinesia [35]. Furthermore, Quan et al. [12] found that hydrogen sulfide can regulate colonic motility by inhibiting both L-type calcium channels and BKCa channels in smooth muscle cells from the rat colon. However, whether the relaxant effect of Evo on rat colon involves direct inhibition of L-type calcium channels is unknown. To investigate this possibility, we examined the effects of Evo on *I*_Ca,L_. We found that Evo could inhibit *I*_Ca,L_. Furthermore, the shape of the I–V curve of *I*_Ca,L_ was markedly changed, suggesting that the voltage gating of L-type calcium channels is modified by Evo. Previous studies suggested that the relaxation of human gallbladder smooth muscle is mainly mediated by ATP-sensitive potassium channel (KATP) [36]. In addition, a number of studies have shown that the activation of K^+^ channels located in the smooth muscle also plays an important role in the relaxant effect on the gastrointestinal smooth muscle mediated by sodium hydrosulfide [37–39]. Whether K^+^ channels are involved in the inhibitory effect of Evo on rat colon motility remains to be studied.

## 5. Conclusions

In summary, Evo inhibits L-type calcium channels in SMCs of rat colon. The relaxant effect of Evo on colonic motility is partly due to direct or indirect inhibition of L-type calcium channels. These data provide the first evidence that Evo may mediate calcium homeostasis in SMCs and therefore play a critical role in regulating colonic motility in rats. However, the role of K^+^ channels in the relaxant effect of Evo on rat colon requires further study.

## Data Availability

The experimental data used to support the findings of this study are available from the corresponding author upon request.
